# Influences of age, mental workload, and flight experience on cognitive performance and prefrontal activity in private pilots: a fNIRS study

**DOI:** 10.1038/s41598-019-44082-w

**Published:** 2019-05-22

**Authors:** Mickaël Causse, Zarrin K. Chua, Florence Rémy

**Affiliations:** 1 ISAE-SUPAERO, Université de Toulouse, Toulouse, France; 2 Université de Toulouse, UPS, Centre de Recherche Cerveau et Cognition, Toulouse, France; 3 CNRS, Cerco, Toulouse, France

**Keywords:** Neural ageing, Cognitive ageing

## Abstract

The effects of aging on cognitive performance must be better understood, especially to protect older individuals who are engaged in risky activities (e.g. aviation). Current literature on executive functions suggests that brain compensatory mechanisms may counter cognitive deterioration due to aging, at least up to certain task load levels. The present study assesses this hypothesis in private pilots engaged in two executive tasks from the standardized CANTAB battery, namely Spatial Working Memory (SWM) and  One Touch Stockings of Cambridge (OTS). Sixty-one pilots from three age groups (young, middle-aged, older) performed these two tasks from low to very high difficulty levels, beyond those reported in previous aging studies. A fNIRS headband measured changes in oxyhemoglobin (HbO2) in the prefrontal cortex. Results confirmed an overall effect of the difficulty level in the three age groups, with a decline in task performance and an increase in prefrontal HbO2 signal. Performance of older relative to younger pilots was impaired in both tasks, with the greatest impairment observed for the highest-load Spatial Working Memory task. Consistent with this behavioral deficit in older pilots, a plateau of prefrontal activity was observed at this highest-load level, suggesting that a ceiling in neural resources was reached. When behavioral performance was either equivalent between age groups or only slightly impaired in the older group, there were not any age-related differences in prefrontal activity. Finally, older pilots with extensive flying experience tend to show better preserved spatial working memory performance when compared to mildly-experienced of the same age group. The present findings are discussed in the frames of HAROLD and CRUNCH theoretical models of cognitive and neural aging, evoking the possibility that piloting expertise may contribute to preserve executive functions throughout adulthood.

## Introduction

In our contemporary society, global population aging results in an increased number of older individuals engaged in complex and risky occupations such as aviation. It is estimated that the majority of current American private pilots are 55 years or older^1^. Accordingly, the potential deterioration of cognitive performance with advancing age becomes an absolute element of concern in this kind of risky activity^2^, probably more than the age-related physical incapacitations^3^. Better knowledge on how age affects specific intellectual functions, which are critical to efficient and safe piloting, would likely help in adapting flying activities to age-related changes in cognitive capacity.

Executive functions are needed during piloting, and there is converging evidence that these functions, such as working memory (WM), spatial attention or inhibition, are affected in healthy aging^4,5^, at least when older individuals must cope with certain levels of task difficulty. Executive functions are known to strongly rely on frontal lobes, frontal-striatal, or frontal-parietal networks^6,7^. The well-documented age-related structural and functional changes in frontal regions likely contribute to executive impairment. Large-scale longitudinal and cross-sectional studies have reported atrophy of the lateral prefrontal cortex^8–10^ and alteration of prefrontal white matter associative tracts^8,11,12^ starting from middle adulthood. Additionally, differences in lateral prefrontal activity in older versus younger adults has been observed^13,14^ and may contribute to age-related changes in performance, although caution is needed as causal relationships cannot be inferred from cross-sectional studies^15^.

To better explore this issue, studies on executive functions that have considered both the effects of age and task load are highly informative. This has been addressed using letter probe WM tasks with variable memory set sizes^7^, using Corsi block-tapping tests with variable sequence length^16^ or using spatial delayed-matching-to-sample tasks with variable difficulty levels^17–19^. According to these studies, older adults demonstrated lower performance relative to young adults for high task loads, whereas performance was equivalent for low task loads^7,16,18^. Moreover, at the lowest task load, prefrontal activity was left-lateralized in younger adults and either increased^16^ or bilateral in older ones^7,18^. Increased dorsolateral prefrontal activity with WM task load was consistently observed in both age groups. Interestingly, in the older group this increase of activity occurred for low task loads, whereas in the younger group the increase occurred for higher task loads^7,16^. At the highest task load, older adults may reach a “capacity limit” as suggested by a plateau in prefrontal activity and concomitant impaired performance relative to younger adults. This set of data overall indicates that depending on the cognitive load in executive tasks, equivalent performance in old and young adults would be associated with prefrontal over-recruitment in old adults, whereas impaired performance in old adults may be associated with either similar or lower levels of prefrontal activity relative to young adults.

Theoretical models of cognitive and neural aging are supported by these age-related changes in executive functioning for variable task demands. The hemispheric asymmetry reduction in older adults (HAROLD) model proposed by Cabeza^20^ postulates that prefrontal activity tends to be less lateralized in older adults than in younger ones. Studies reporting age effects on prefrontal activations during WM tasks seem to support this model when task demands are low^7,17,18^. The latter studies suggest that older participants’ bilateral prefrontal recruitment may be beneficial to WM performance. Rather than the result of dedifferentiation, i.e., an age-related decrease in functional specialization of brain regions^21^, supplementary brain activity in contralateral hemispheres would serve to compensate for reduced efficiency in regions implicated in the task at hand. However, the HAROLD model seems not well supported by experimental data when task loads are high^7,17,18^. To account for load effects, the Compensation-Related Utilization of Neural Circuits Hypothesis (CRUNCH) model has been hypothesized^22^. This model predicts that more cortical regions would be activated as task load increases. Accordingly, age-related compensatory activation can be efficient at lower levels of task difficulty, but as demands increase, a resource ceiling might be reached, leading to insufficient processing and a decrement in performance relative to young adults. Thus, older adults would progress from over-activation at lower levels of task demand to under-activation at higher levels of task demand, due to a resource ceiling. Young adults would also reach a resource ceiling, but for even higher task demands^7^. The CRUNCH model is supported by previous results assessing WM performance for different task loads and comparing young and older groups^7,17–19^. However, several life course factors are thought to play a major role in cognitive and neural aging, especially regarding efficiency of compensatory mechanisms in older adults^23^. Piloting can be seen as a cognitive training since it engages a high amount of tasks, which places strong demands on spatial attention, WM and planning^24^. Among others, pilots must control the aircraft, apply numerous procedures and rules, pay a sustained attention to aircraft spatial position, process continuously ongoing events to maintain an up-to-date situation awareness^25,26^, interpret numerous instruments, plan actions and decisions over time, adapt to changing contexts etc. The relationship between age and piloting performance has been found to be mediated by a cluster of cognitive factors, among which WM plays an important role^27,28^. WM has been shown to correlate with flying performance in several other studies e.g.^25,29^. Also, reasoning/problem solving is predictive of aeronautical decision-making relevance, in particular in novice pilots^30^, and spatial cognition is considered at the heart of the building of pilots’ situation awareness^31,32^. Therefore it remains open whether middle-aged and older pilots exhibit patterns of performance and neural activity similar to those predicted by CRUNCH and HAROLD models, during spatial WM and planning tasks. Addressing this question will help assessing possible increased risk in older versus younger pilots. Moreover, it may contribute to evaluating the potential benefits of piloting on the preservation of executive functions at old age.

The objective of the present study was twofold: first, we aimed to investigate age-related changes in executive performance in a population of pilots, in order to evaluate potential increased risk with age. Second, we aimed to relate the observed changes in older pilots with current theoretical models of cognitive and neural aging^22,23,33,34^. To this end, cognitive performance and prefrontal brain activity were examined in private pilots from three different age groups (young, middle-aged, older) owning variable flight experience. Since piloting likely requires executive functions^27,28^, participants were tested on two executive tests from the CANTAB battery^6^ assessing visuospatial WM, and planning/reasoning. Visuospatial working memory was evaluated with the Spatial Working Memory (SWM) task. Planning/reasoning was evaluated with the One Touch Stockings (OTS) of Cambridge task. It is worth noting that we manipulated task difficulty from low to very high levels, i.e. beyond the ones explored in previous protocols using CANTAB, such as in a study aiming at developing normative CANTAB test performance over the life span^35^. Testing very high levels of difficulty was a critical point, as we assumed that pilots develop and maintain higher cognitive performance relative to the general age-matched population. Across the three different age groups, we examined the effects of task difficulty on performance and prefrontal brain activity as measured with fNIRS. The fNIRS technique has been successfully used in previous studies to assess variations in brain activity due to aging^36,37^ or due to variation of mental workload e.g.^38–41^. We hypothesized that, in all age groups, increased mental workload (resulting from higher task difficulty) would negatively impact performance and induce increased activity in the lateral prefrontal cortex, as predicted by the CRUNCH model. Also we investigated whether, at low task difficulty, older participants would demonstrate more pronounced increase of prefrontal activity when compared to younger ones, possibly reflecting compensatory strategies to maintain performance^7^. At higher levels of difficulty, we hypothesized that asymptotic prefrontal activity would be reached, reflecting a “processing capacity limit”. We specifically investigated whether this capacity limit would be reached from lower difficulty levels in older vs. younger pilots, and whether flight expertise of older pilots could influence this capacity limit.

## Results

### Spatial working memory (SWM) task

#### Task performance

**Figure 1:**
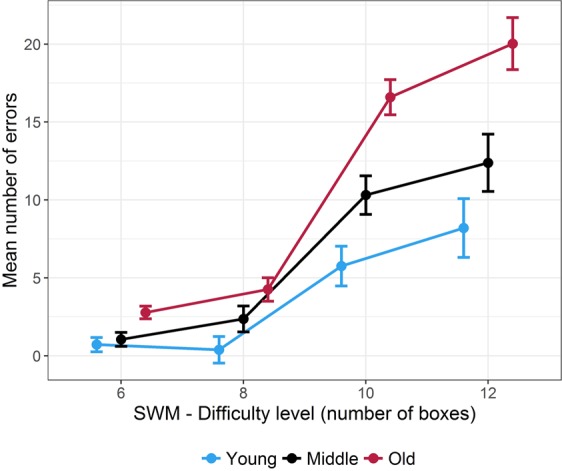
Spatial Working Memory (SWM) performance. Mean number of errors with respect to age group across the four levels of difficulty. Error bars represent the standard error of the mean.

Mean and s.e.m. for number of errors are shown in Fig. 1. All values are provided in Supplementary Table S1. Mean response latencies were: 6 boxes = 28.30 s (SD = 10.01 s); 8 boxes = 43.18 s (SD = 23.52 s); 10 boxes = 74.08 s (SD = 23.75 s); 12 boxes = 89.21 s (SD = 25.08 s), irrespective of the age groups. The mean number of errors was increased with respect to both *difficulty* and *age group* (*F(*3, 171) = 119.70, *p* < 10^−6^, ^η^_*p*_^2^ = 0.68; *F(*2, 57) = 19.29; *p* < 10^−6^, ^η^_*p*_^2^ = 0.40, respectively). The number of errors was significantly higher for 10 vs. 8 boxes (*p* < 0.05, FDR corrected), and for 12 vs. 10 boxes (*p* < 0.05, FDR corrected). Older group committed more errors than the younger one (*p* < 0.05, FDR corrected). The two-way interaction (*difficulty* × *age group*) was also significant (*F(*6, 171) = 7.03, *p* < 10^−6^, ^η^_*p*_^2^ = 0.20). The three age groups had equivalent task performance for 6 and 8 boxes. In the two highest level of difficulty (10 and 12 boxes), middle-aged participants committed more errors than younger ones (*p* < 0.05, FDR corrected, in both comparisons) and older participants committed more errors than young and middle-aged ones (*p* < 0.05, FDR corrected, in all comparisons).

#### Cerebral hemodynamics

**Figure 2:**
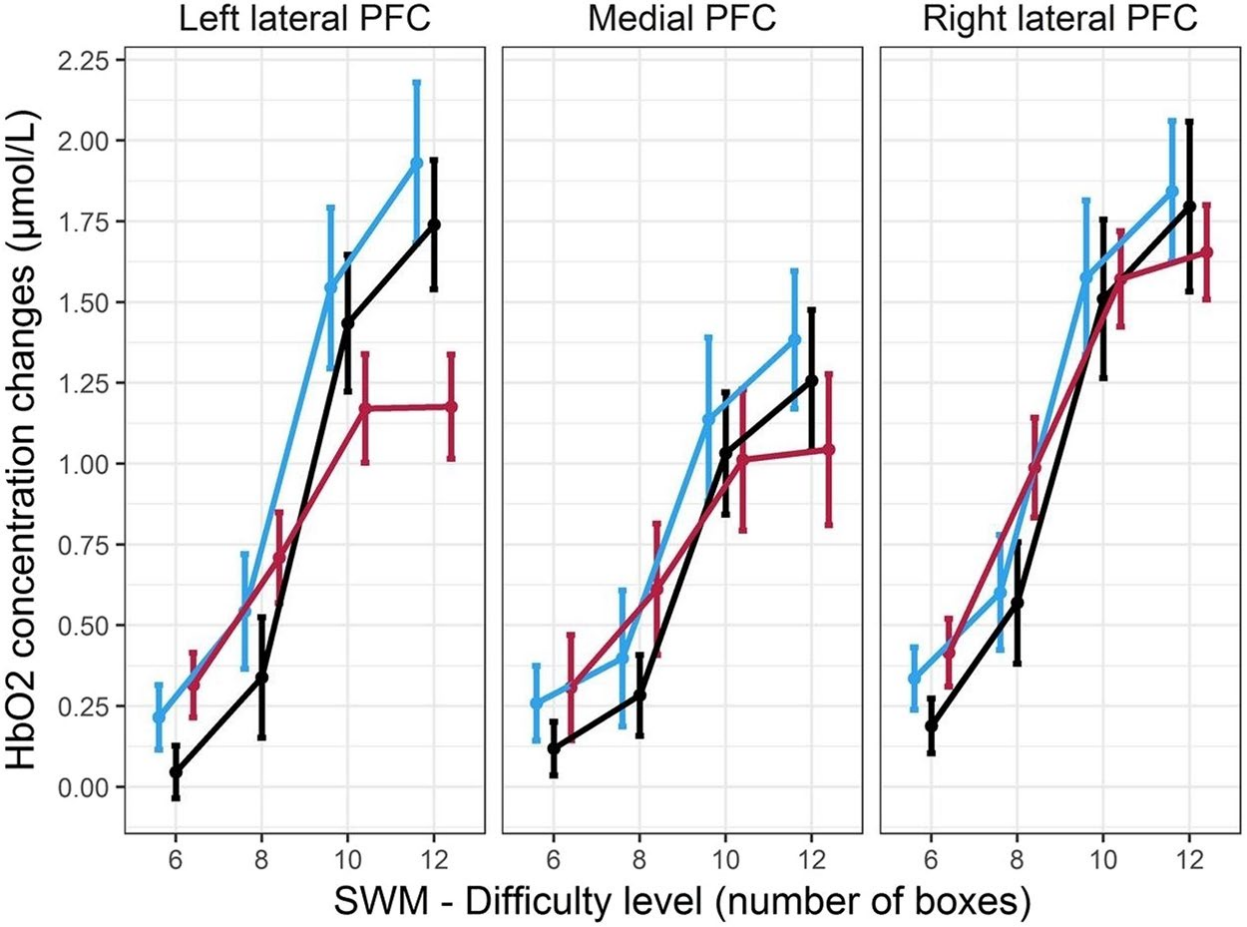
Mean HbO2 concentration changes when compared to rest (µmol/L) in the 3 prefrontal regions of interest, with respect to age groups across the four levels of difficulty of the Spatial Working Memory task (SWM). Increased difficulty generated an increase of HbO2 concentration in the three groups. A plateau of activation was observed between the two highest levels of difficulty (10 and 12 boxes) in the older group, within the left prefrontal cortex. Increased HbO2 concentration was more pronounced in the lateral regions. Error bars represent the standard error of the mean.

**Figure 3:**
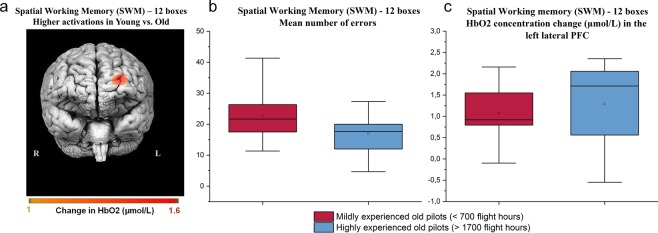
(**a**) Topographic image generated via fNIRSoft^84^ showing positive concentration changes in HbO2 concentration in young vs. old group during the highest level of difficulty of the Spatial Working Memory task (SWM). Younger participants showed significantly stronger activations in the left PFC relative to older ones. (**b**) Mean number of errors in mildly- and highly-experienced old pilots during the highest level of difficulty of SWM. c. HbO2 concentration change in the left PFC in mildly- and highly-experienced old pilots during the highest level of difficulty of SWM. In (**b**,**c**), the square dots represent mean values in subgroups, the horizontal lines represent median values, the boxes represent inter-quartile ranges (25–75% percentiles), and the whiskers represent the minimum and maximum values.

Mean and s.e.m. for HbO2 concentration changes are shown on Fig. 2. Topographical map of the change in HbO2 concentrations are shown in Supplementary Fig. S1. All values for HbO2 concentration changes are provided in Supplementary Table S2. The main effect of *difficulty* on prefrontal HbO2 was significant (*F(*3, 171) = 114.03, *p* < 10^−6^, ^η^_*p*_^2^ = 0.68), with an increase of HbO2 concentration for 8 vs. 6 boxes, 10 vs. 8 boxes (*p* < 0.05, FDR corrected, in both comparisons), but not between 12 vs. 10 boxes (*p* > 0.05, FDR corrected). There was also a main effect of *ROI* (region of interest) (*F(*2, 114) = 11.99, *p* < 10^−4^, ^η^_*p*_^2^ = 0.17). HbO2 concentration was higher in the left and right lateral prefrontal cortex (PFC) than in the medial region (*p* < 0.05, FDR corrected, in both comparisons) and higher in the right PFC than in the left PFC (*p* < 0.05, FDR corrected). Furthermore, there was a significant interaction between *difficulty* and *age group* (*F(*6, 171 = 3.26, *p* = 0.005, ^η^_*p*_^2^ = 0.10), with an HbO2 increase (irrespective of the *ROI*) across the two lowest difficulty levels (i.e. 8 vs. 6 boxes) in the older group only (*p* < 0.05, FDR corrected). The interaction between *difficulty* and *ROI* was also significant (*F(*6, 342 = 12.17, *p* < 10^−6^, ^η^_*p*_^2^ = 0.28), with lateral prefrontal HbO2 concentration being more important than in the medial region, in particular during the two highest difficulty levels (*p* < 0.05, FDR corrected, in all comparisons), whereas HbO2 concentration in lateral and medial regions was equivalent at the easiest level of difficulty (i.e. 6 boxes) (*p* > 0.05, FDR corrected, in both comparisons). In the 8 boxes condition, only the right lateral PFC showed higher HbO2 concentration level than the medial PFC (*p* < 0.05, FDR corrected). Finally, the three-way interaction *difficulty* × *ROI* × *age group* was significant (*F*(12, 342) = 1.83, *p* = 0.041, ^η^_*p*_^2^ = 0.06). Post–hoc tests revealed that, while young and middle-aged participants showed increased left prefrontal activity across all consecutive levels of difficulty, i.e. 8 vs. 6 boxes, 10 vs. 8 boxes, and 12 vs. 10 boxes (*p* < 0.05, FDR corrected, in all comparisons), the older participants showed this increase in left prefrontal activity only across the three first levels of difficulty, i.e. 8 vs. 6 boxes and 10 vs. 8 boxes (*p* < 0.05, FDR corrected, in all comparisons). Then a plateau of activity was observed, as left PFC HbO2 concentration was unchanged between 10 and 12 boxes (*p* > 0.05, FDR corrected), see Fig. 2. As a consequence, younger participants had higher left PFC activity than older ones in the most difficult condition (12 boxes) (p < 0.05, FDR corrected) (Fig. 3a). Main effect of *age group* and *age group* × *ROI* interaction effect were not significant (F < 1, *p* > 0.05 for both effects).

Since age-related differences in both performance and brain activity were specifically evidenced in the 12-boxes condition, we further investigated for this highest difficulty level whether extensive piloting experience in older participants could be associated with better preserved performance and higher prefrontal activity. We found that highly-experienced old pilots (>1700 flight hours) had higher performance than less experienced pilots (<700 flight hours) in the 12-boxes SWM task (one-tailed t-test, *p* = 0.045), see Fig. 3b. There was no significant effect of flying experience on HbO2 concentration in the left PFC (one-tailed Mann-Whitney U test, p > 0.05), see Fig. 3c.

### One touch stockings (OTS) of cambridge task

#### Task performance

**Figure 4:**
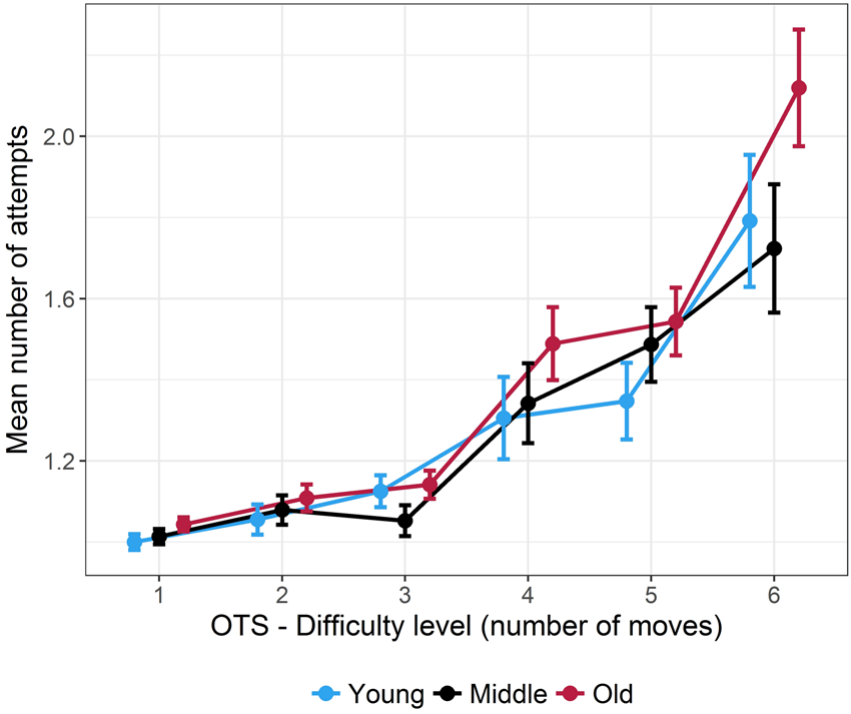
One Touch Stockings (OTS) of Cambridge task performance. Mean number of attempts with respect to age groups across the six levels of difficulty. Error bars represent the standard error of the mean.

Mean and s.e.m. for number of attempts are shown in Fig. 4. All values are provided in Supplementary Table S3. Mean response latencies were: 1 move = 5.76 s (SD = 3.59 s); 2 moves = 7.22 s (SD = 5.32 s); 3 moves = 8.26 s (SD = 4.20 s); 4 moves = 18.10 s (SD = 13.45 s); 5 moves = 44.19 s (SD = 37.05 s); 6 moves = 62.09 s (SD = 38.72 s). The mean number of attempts increased with *difficulty* (*F(*5, 285) = 46.77, *p* < 10^−6^, ^η^_*p*_^2^ = 0.45). The number of attempts was significantly higher for 6 moves when compared to all lower levels of difficulty (*p* < 0.05, FDR corrected, in all comparisons), for 5 moves vs. 1, 2 or 3 moves (*p* < 0.05, FDR corrected, in all comparisons), and for 4 moves vs. 1, 2 or 3 moves (*p* < 0.05, FDR corrected, in all comparisons). Moreover, the number of attempts significantly increased across *age groups* (*F(*2, 57) = 3.45, *p* = 0.038, ^η^_*p*_^2^ = 0.11). The older group committed more errors than the young and middle-aged groups (*p* < 0.05, FDR corrected, in both comparisons). Number of attempts in young and middle-aged groups did not differ significantly (*p* > 0.05, FDR corrected). The interaction between *difficulty* and *age group* was not significant (*F(*10, 285) = 1.03, *p* = 0.350, ^η^_*p*_^2^ = 0.04).

#### Cerebral hemodynamics

**Figure 5:**
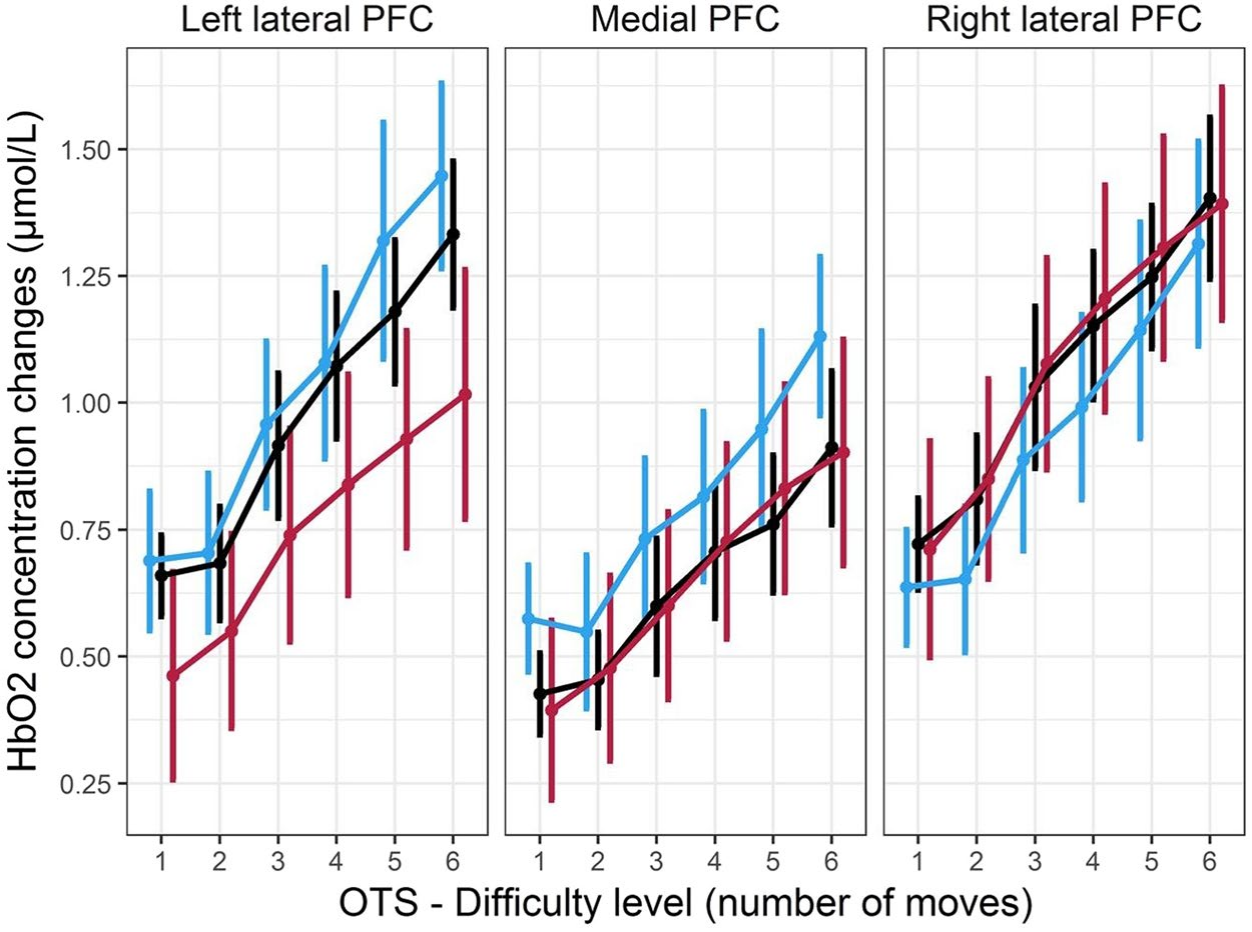
Mean HbO2 concentration changes relative to rest (µmol/L) in the 3 prefrontal regions of interest, for the different age groups across the six levels of difficulty of the One Touch Stockings (OTS) of Cambridge task. Increasing difficulty generated an overall increase of the HbO2 concentration in the three groups. HbO2 concentrations were more important in the lateral vs. medial parts of the PFC. Error bars represent the standard error of the mean.

Mean and s.e.m. for HbO2 concentration changes are shown on Fig. 5. Topographical map of the change in HbO2 concentrations are shown in Supplementary Fig. S2. All values for HbO2 concentration changes are provided in Supplementary Table S4. The HbO2 concentration significantly increased with *difficulty* (*F(*5, 285) = 78.70, *p* < 10^−6^, ^η^_*p*_^2^ = 0.58) with a significant difference across all consecutive difficulty levels (*p* < 0.05, FDR corrected, in all comparisons) with the exception of 2 vs. 1 move comparison (*p* > 0.05, FDR corrected). The effect of *ROI* was also significant (*F(*2, 114) = 12.50, *p* < 10^−4^, ^η^_*p*_^2^ = 0.18) with left and right PFC showing greater HbO2 concentrations than the medial region (*p* < 0.05, FDR corrected, in both comparisons). The interaction between *difficulty and ROI* was also significant (*F(*10, 570) = 5.13, *p* < 10^−6^, ^η^_*p*_^2^ = 0.08), with the HbO2 concentration increase due to increasing difficulty being more pronounced in lateral prefrontal regions than in the medial region. All other main and interaction effects (i.e. *age group*, *ROI* × *age group*, and *difficulty* × *age group)* were not significant (Fs < 1 *ps* > 0.05).

## Discussion

Aging of the general population results in an increased engagement of older individuals in risky activities. This evolution of the society calls for an improved understanding of changes in cognitive performance and related neural activity with respect to age, in particular when task demand increases. Under this objective, sixty-one private pilots from three age groups completed two standardized laboratory tasks that were designed to engage Spatial Working Memory (SWM, four levels of difficulty) and strategic planning and organization of behavior towards a goal (OTS, six levels). fNIRS was used to record variations in HbO2 concentrations in the prefrontal cortex.

As hypothesized, in the three age groups, mean number of errors in the SWM task was increased as a function of task difficulty. For low and moderate levels of difficulty (up to 8 boxes), there was not any effect of age on the number of errors. At highest difficulty levels (10 or 12 boxes), older and middle-aged participants committed more errors than younger participants. Consistently, several studies have reported age effects on committed errors in spatial WM tasks, with marked impairment in older individuals for the highest tested difficulty levels^6,35,42^. However, age-related differences in performance have been generally observed from lower WM demands when compared to our study. Using an identical SWM task from the CANTAB battery, higher number of ‘between-search errors’ in older relative to young adults has been reported from 6- to 8-box levels of difficulty^6,35^ (note that greater numbers of boxes have not been tested in these two works). The latter studies also used ascending orders of difficulty when testing participants, making their results highly comparable to the present ones^42^. Our results show that for previously tested levels of difficulty of the SWM task, middle-aged and older pilots did not show degraded performance relative to young ones. This pattern of performance as a function of age and task demand is very similar to the pattern previously reported in selected young and older “high performers” (defined a best performers in their own age group) on a different spatial WM task^43^. Indeed our sample of older pilots may represent selected individuals, with high education level and efficient spatial skills^44^. However, our results show that in pilots, an age-related impairment of spatial WM function is still observed but it appears at very high levels of task complexity.

Interestingly, in the highest difficulty level of the SWM task, our older pilots with extensive lifelong flying experience (more than 30 years of license and more than 1700 flight hours) tend to show better preserved performance when compared to mildly-experienced of the same age group. It has been proposed that life course factors such as long-term engagement in social or intellectual activities may positively impact cognitive function in older adults. It is possible that high levels of practice could allow maintenance of proficiency in specific trained skills^23^. In particular, preserved spatial abilities with age may have resulted from extensive flight experience^45^. This latter result based on two small sub-samples has to be confirmed with larger groups. Also, even if previous results^6,35,42^ reported with the same battery of tasks on non-pilots served as our reference, a future study should compare aging effects between pilots and non-pilots, the latter participants acting as a control group. This would help draw stronger conclusions about positive impact of piloting on preservation of executive functions. Finally, several-year longitudinal studies assessing effects of aircraft piloting experience on cognition and brain activity during laboratory executive task performance would be particularly relevant to confirm this hypothesis.

fNIRS data showed that, in the three age groups, increased difficulty on the SWM task induced an increase of HbO2 concentration, more specifically in the lateral regions of the prefrontal cortex. This result is consistent with previous fMRI studies investigating WM load effects on dorsolateral prefrontal activity^46–49^ and confirms the reliability of fNIRS to track variations in mental workload^38–41^. Notably, patterns of load-modulated activity were different in the older group, with a plateau observed between the 10- and 12-box levels of difficulty. This suggests a WM “processing capacity limit”^7^ in the older group, which was not reached in the middle-aged and young groups, at least for the tested difficulty levels. Our results in the CANTAB SWM task can be compared with those from previous aging studies^7,18^ using spatial delayed-matching-to-sample, spatial n-back, or Corsi block-tapping tasks. In that preceding works and for low levels of difficulty, older participants evidenced bilateral prefrontal activity whereas activity was unilateral in younger participants, along with equivalent performance^7,18^, for meta-analysis, see^50^. Preservation of spatial WM performance was similarly observed in our SWM task up to a moderate difficulty level (8 boxes). We did not find any significant difference in prefrontal activity between the three age groups for these low to moderate WM loads, although the CRUNCH^22^ model would predict higher activity in older participants. Again, pilots may represent selected individuals with better preserved cerebral function^51^. Alternatively, it is possible that the low spatial resolution of fNIRS (compared for fMRI) did not enable detecting some changes in lateral prefrontal activity. Indeed, age-related lateral prefrontal over-activation during low-load WM tasks has been specifically reported in the inferior frontal gyrus (Brodmann areas 44/45) in previous fMRI studies^7,18^. Activity differences in small-extent regions may have not been detected.

At very high level of difficulty (12 boxes), older participants showed lower left prefrontal activity than younger participants, along with impaired performance. This is consistent with previous results using different spatial WM tasks^7,16,18^ and with predictions from CRUNCH^22^. It has been proposed that, for high demands, older individuals show reduced accuracy because the task might be approached with ineffective strategies or because older adults partly disengage from it, which can lead to under-activation compared to younger individuals^52^. Notably, in our study, age-related under-activity was observed for a very high difficulty level. This indicates that, in older pilots, a resource ceiling was only reached for the highest WM load. This ceiling likely reflected individuals’ WM capacity limit^7^ and drove performance on the SWM task. It remains open whether, and in which real-life flying conditions, this particular deficit may impact aircraft control and decision making in elderly vs. young pilots. Aviation is a multi-tasking environment^53^ that inherently involves high demands on spatial attention and WM. These functions are particularly relevant for simultaneous on-line monitoring of the various flight parameters and crucially needed when processing ongoing events to continuously adjust flight path^54^.

To summarize, results observed in the SWM task did not support the HAROLD model (i.e. we did not observe less asymmetric activations in older pilots) and supported to some extent the CRUNCH model. Younger pilots showed a continuous increase of the hemodynamic response with increased task difficulty. In contrast, a less linear pattern of activation was found in the older pilots. Indeed, our fNIRS data evidenced a plateau of prefrontal activation from 10- to 12-box levels, in the older group exclusively. As a consequence, older pilots showed under-activation at higher levels of task demand when compared to younger pilots, likely due to a resource ceiling according to the CRUNCH model. However, activity was not significantly different between age groups in low difficulty conditions, while the CRUNCH model would rather predict higher activation in older relative to younger groups. This absence of over-activation in older pilots likely reflects a preservation of spatial working memory performance at moderate difficulty level. It is worth noticing that older pilots’ decrease of performance and plateau of activation for the highest level of difficulty of SWM may also have reflected motivational factors. According to the motivational intensity theory^55^, resources are important for survival, so individuals tend to avoid wasting them and aim at recruiting only those that are required to successfully perform a task. Naturally, more difficult tasks require higher amount of resources, and if one invests effort in a very difficult task where success is impossible, resources would be wasted. Thus, one cannot exclude that older pilots disengaged from the SWM in the highest level of difficulty because they perceived lower success likelihood than their younger counterparts. In this sense, the disengagement may have reflected resource saving behavior. In addition, older pilots may have been less motivated to perform very difficult abstract cognitive tasks, or an accumulation of fatigue (difficult trials were at the end of the task) could also have contributed too poorer performance at highest levels of demand. However, motivation and fatigue were certainly not the only factors that produced a disengagement from the SWM task, otherwise we assume that such effect would have been also observable in the OTS task, in which overall difficulty was also very high (in particular in the 5 and 6 move conditions).

In the OTS task, Tower of Hanoi-variant task assessing spatial planning and reasoning, the older group evidenced an overall increased number of attempts prior to correct answer, when compared to younger groups. The age effect size was relatively small showing that age explained only a limited part of the variance of the data. Variations in performance across difficulty levels were not different between the three groups. Using the CANTAB three-ball Tower of London (TOL) task identical to our OTS task, a significant age-related impairment (defined as a reduced percentage of trials completed in the minimum number of moves) was observed in the 3- and 5-move conditions^35^ or only in the 3-move condition^6^. Note that the 6-move task was not assessed in these studies. Aging effects on TOL or three-disk Tower-of-Hanoi performance with varying difficulty have therefore not been consistently described, and may in particular depend on learning effects across consecutive trials^56–58^. Nevertheless, a reproducible finding is that spatial planning was found to be mildly impacted in older adults (at least below 70 years of age), when compared to other executive components such as WM. In our study, increased OTS difficulty induced an increase in prefrontal activity. This is consistent with previous fMRI results from Newman, Carpenter, Varma, and Just^59^, and Brennan, Welsh, and Fisher^56^ who reported increased frontal and parietal activity from easy to difficult levels (1 to 6 moves) in the Tower of Hanoi task. Our data did not show any age-related differences in prefrontal activity, with all age groups evidencing a quasi-linear increase of activity as a function of task load. As underlined above, the influence of age on task performance was smaller in the OTS than in the SWM task (effect size, ^η^_*p*_^2^ = 0.12 vs. ^η^_*p*_^2^ = 0.41, respectively). It is well known that cognitive functions are not all impacted simultaneously during aging^60^. The relatively lower impact of age on OTS performance, with no age × difficulty interaction, may explain the absence of age effect on prefrontal activity and the absence of any plateau of activity, suggesting that resources ceiling was not reached for this task in any of the groups. Newman *et al*.^61^ highlighted that the TOL task recruits right and left prefrontal cortex, respectively involved in planning generation and execution, but also the left and right superior parietal cortex, that may, along with frontal areas, subserve visuospatial attentional processes. Attentional control may be less efficient with age^7^. Rönnlund, Lövdén, & Nilsson^62^ showed that the deficit observed with age in a Tower of Hanoi-type task was not only explained by a deficit in frontal lobe executive functions, but also by a decrease in visuospatial skills, the latter being largely supported by regions outside the frontal lobe (in particular the parietal regions). Therefore in our study, the limitation of fNIRS measurements within the prefrontal cortex may explain that the slight age-related decline in OTS performance was not accompanied with any significant alteration of prefrontal activity.

## Conclusion

This fNIRS study confirmed that prefrontal activity tends to progressively increase as a function of task difficulty. It also demonstrates anew the existence of a general cognitive impairment related to age. Besides, we bring further evidence that cognitive load accentuates the age-related deficit in a Spatial Working Memory (SWM) task. This impairment may occur due to a ceiling in neural resources. In both tested executive tasks, our data do not provide convincing evidence for compensatory prefrontal activity in older relative to younger pilots. Our results rather suggest that, for moderate levels of cognitive load, both performance and associated neural mechanisms would be preserved in older pilots. This brings further knowledge on how trained older individuals may be able to efficiently engage similar neural resources as their younger counterparts, at least up to certain task load levels, thereby maintaining executive function at high performance levels. In this sense, in the highest task load levels, older pilots with extensive flying experience tend to show better preserved spatial working memory performance when compared to mildly-experienced of the same age group.

Since aircraft piloting typically engages high-level cognitive control, our results underline the importance for developing onboard systems that avoid excessive demands on working memory. Piloting expertise may contribute to maintenance of cognitive performance, therefore routine cognitive tests are recommended as an alternative to the use of chronologic age when determining a person’s ability to perform at work^63^. Assessing spatial working memory skills through a wide range of demand levels would be also desirable at the pilots’ selection stage, as this cognitive ability has been shown to correlate with training success^64^. More generally, assessing cognitive performance can help anticipating later effects of aging on flying activity, pilots with higher initial cognitive performance are better protected against aging effects^65^, as their piloting performance is less impacted by age. Moreover, developing training methods to improve cognitive and neural efficiency^66^ or virtual assistants that provide an extra memory are promising ways in order to allow older individuals to continue performing risky activities with the best level of safety. Finally, including fNIRS or similar methods can be relevant when assessing pilots’ ability to perform as they get older. Along with evaluation of cognitive performance, fNIRS data may represent a reliable estimate of pilots’ mental effort^39^. Such a measure of cerebral activity can bring additional fine-grained information on one’s amount of brain resources recruited to reach a given performance (i.e., neural efficiency).

## Method

### Participants

Sixty-one highly educated pilots aged from 19 to 74 years, were recruited for this study. None of them reported a history of psychiatric or neurological disorder. All were French private aviation pilots (otherwise referred to as “private pilots”). The study complied with the Declaration of Helsinki for human experimentation and was approved by regional Ethics committee (Comité de Protection des Personnes du Sud-Ouest et Outre-Mer IV, n°CPP15-010b/2015-A00458-41). All participants provided written informed consent. Participants belonged to one of the following age groups: young (19–25 years), middle-aged (30–48), and older (51–74). There were 18 young (m = 21.0 ± 1.6 years, 1 woman), 19 middle-aged (m = 38.3 ± 6.9 years, 2 women), and 24 older (m = 62.3 ± 6.6 years, 0 women) participants. It has to be noticed that our three age groups were almost exclusively male, although representative of French pilots’ population. The average level of education was high, with 14.7 years (±1.2 years) of education in the young group, 16.6 years (±1.3 years) in the middle-aged group, and 15.9 years (±2.6 years) in the older group. The total flight experience was of 48.9 hours (±57.0 hours) in the young group, 938.4 hours (±1603.3 hours) in the middle-aged group, and 3848.7 hours (±5610.0 hours) in the older group. Based on total flight experience, older adults belonged to either “highly-experienced” or “mildly experienced” subgroups. The highly-experienced older pilots (n = 12) all had over 1700 flight hours (mean: 7276 h, ±6332 h). Moreover they all had their license for more than 30 years (mean: 43 years, ±7.2 years). The mildly-experienced older pilots (n = 12) all had less than 700 flight hours (mean: 420 h, ±258 h). They had their license for 22 years on average (±16.4 years). Age was equivalent in the two subgroups (*p* > 0.05), with 63.1 ± 6.8 years in the highly-experienced subgroup and 61.4 ± 6.8 years in the mildly-experienced one. Level of education was also similar in both subgroups (highly-experienced: 16.3 ± 3.0 years; mildly-experienced: 15.3 ± 2.3 years, *p* > 0.05).

### Experimental procedure and tasks

The fNIRS device was installed on the participants’ forehead before starting the experiment. A short training session was proposed before each task, consisting of instructions and several practice trials, in accordance to the CANTAB software manual. More specifically, participants first received instructions at the beginning of each task, during a first “demonstration trial”, meaning that instructions were given while the experimenter actually showed how to perform the task. Then, participants performed a few practice trials to ensure that they correctly understood the principle of each task (see below). The training lasted approximatively 2.5 minutes for each task, with slight variations depending on participants. Average task completion (excluding instruction/training) was approximatively 9 minutes for SWM and 11 minutes for OTS. It took approximately 30 minutes to complete both tasks (including instructions, training, and a short break between the two tasks).

### Spatial working memory (SWM) task

**Figure 6:**
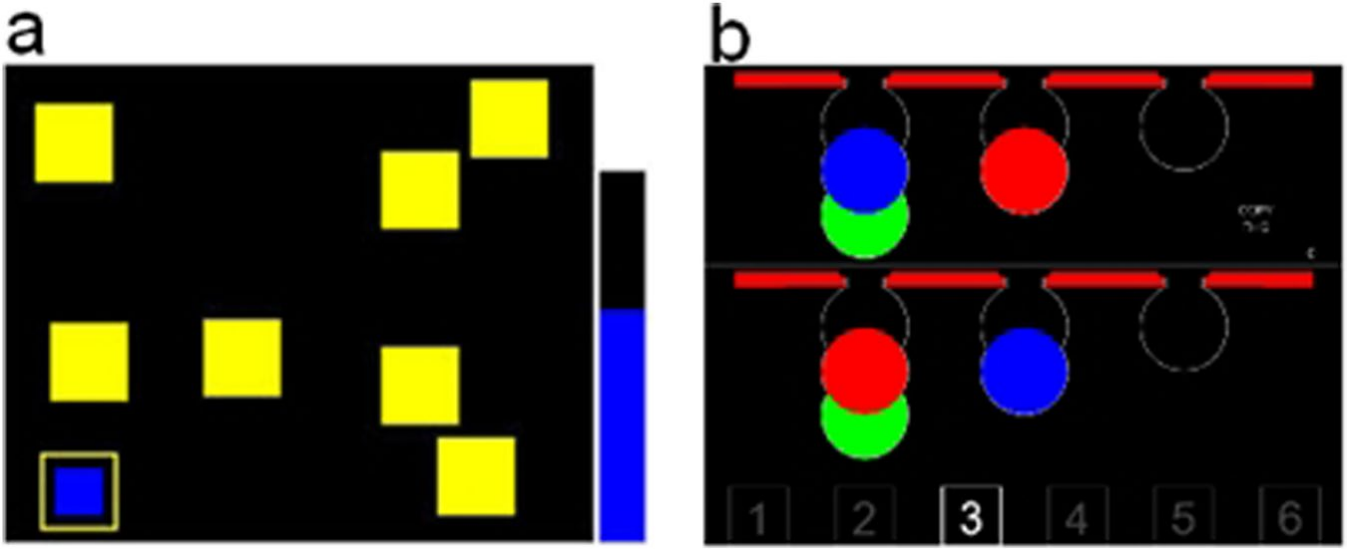
CANTAB Spatial Working Memory (SWM) (**a**) and One Touch Stockings (OTS) of Cambridge (**b**) tasks. In this example, the SWM level of difficulty is 8 boxes and the OTS level of difficulty is 3 moves.

This task involves retention and manipulation of visuospatial information in working memory. The rationale for this task and its implementation has been described previously in some detail^67^. Participants were required to search through a number of colored boxes by touching each one in order to “open it” and thus revealing its contents. The goal was to find and collect blue tokens hidden inside the boxes and, once found, to use them to fill an empty column at the right side of the screen (Fig. 6a). Participants were instructed that once a token was found in a box, that box would not hide another token during the current trial. Thus, the total number of tokens was equal to the number of boxes, and only one token was hidden in a given box during a trial. Before starting the task, there were 1 demonstration trial and 2 practice trials. The demonstration trial showed an example with 3 boxes. Then, participants completed two practice trials (3 boxes also). We used the “high functioning” mode of the task, which included four levels of difficulty (6, 8, 10, 12 boxes) for a total of eight trials without any break: 6, 8, 10, 10, 10, 12, 12, 12 boxes. There was a 3 s inter-trial interval between each trial. Task performance was measured as the number of times the participant opened a box, in which a blue token was already found in a previous search of the current trial (also referred as “between-search errors” in previous studies that have used identical CANTAB task^6,35^. Lower values indicate better performance.

### One touch stockings (OTS) of cambridge task

This task is close to the Tower of London task, which provides a simpler version of the Tower of Hanoi problem^68^. The computerized version has been described in detail by Owen *et al*.^67^. To summarize the principle, at the beginning of each trial, two sets of colored balls were presented in predefined positions, one in the top half of the screen and one in the bottom half. These were described as pool balls, since they appear to be hanging in pockets (Fig. 6b). Participants were asked to determine the minimum number of moves necessary to rearrange the three balls in the bottom display, such that their positions matched the goal arrangement in the top half of the screen. As in the Tower of Hanoi, balls below other balls cannot be moved without first moving the balls above. Participants were asked to press the button with the number (1 to 6) that corresponded to the minimum number of moves needed to match the goal arrangement. Before starting the task, there were 1 demonstration trial and 3 practice trials. As for the SWM task, the rules were explained during the first demonstration trial showing an example of the 1 move condition. Then, participants completed three practice trials (2, 2, and 3 moves, respectively). We used the “high functioning” mode of the task, consisting of six levels of difficulty (1, 2, 3, 4, 5, 6 moves) for a total of twenty four trials, with difficulty levels pseudo-randomized across trials. There was a 3 s inter-trial interval between each trial. Task performance was measured as the mean number of attempts (also called “mean choices to correct”) until the correct answer was achieved. Accordingly, values above 1 indicate errors and lower values reflect better performance.

### fNIRS data acquisition

**Figure 7:**
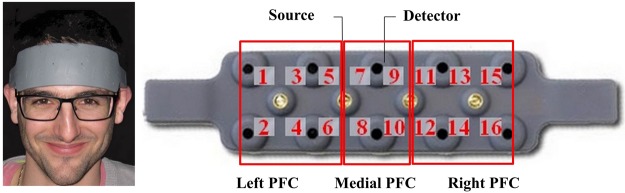
Biopac fNIR 100 optode configuration and illustration of the 3 prefrontal ROIs used in the statistical analysis. Image adapted from^84^.

Participants were asked to perform the SWM and the OTS tasks with the fNIRS equipment (Biopac fNIR 100, Fig. 7) attached to the forehead. The 16-optode fNIR 100 system records data at 2 Hz and measures changes in HbO2 and HHb (both in *µ* mol/L, using the modified Beer-Lambert Law) with two peak wavelengths at 730 nm and 850 nm. The fNIR 100 has a fixed 2.5 cm source-detector separation, see Fig. 7 (please note that the participant visible on this figure gave its informed consent for publication of identifying images in an online open-access publication). The differential pathlength factor (DPF), which accounts for the increased distance travelled by light due to scattering, was set at 5.76. This value is in the recommended range for an adult head^69,70^. According to the literature, DPF is age-dependent^71^. While we kept the same DPF value for all our participants, it would be desirable to adapt the DPF according to the participant age in a future study, for example using the general equation provided by Scholkmann & Wolf ^72^. COBI Studio (v1.2.0.111) and fNIRSoft (v1.3.2.3), both Biopac software, were used for data acquisition and analysis respectively. Topographical maps shown in Supplementary Material (Supplementary Figs S1 and S2) were also generated using fNIRSoft (v1.3.2.3). Before each executive task, participants were asked to relax for approximately two minutes, and a ten-second baseline measurement was then performed. Changes in HbO2 and HHb concentrations from this ten-second rest period baseline were computed^38,40,73^. To remove long-term drift^74^, higher-frequency cardiac or respiratory activity and other noise with other frequencies than the target signal^75–78^, we used a band-pass FIR filter with an order of 20 (0.02–0.40 Hz) on this raw time series of HbO2 and HHb signal changes. After this process, a correlation-based signal improvement (CBSI method^74^) algorithm was used to filter out movements artefacts and spikes and to improve signal quality based on the assumed negative correlation between HbO2 and HHb^79^. A side-effect of CBSI method is that it “forces” HbO2 and HHb signals to be inversely correlated. Therefore, it is somewhat artificial to report both HbO and Hbb since CBSI makes them almost mirror-images of one another. In this, respect we only reported the corrected HbO2 signal, otherwise called “corrected activation signal” in Cui *et al*.^74^. HbO2 concentrations changes were then averaged across all trials for each condition. More precisely, changes in HbO2 concentrations from the ten-second rest period baseline were computed over the average concentration of the combined trials of each condition. For the computation of the average of each trial value, the entire trial duration was used. Based on previous literature using the same fNIRS device^39,80,81^, statistical analysis was focused on 3 ROIs: left lateral PFC (optodes 1–6), medial PFC (optodes 7–10), and right lateral PFC (optodes 11–16).

### Statistical analysis

Two-way repeated-measures ANOVAs were used to analyze numbers of errors in the two CANTAB tasks, with within-subjects factor of task *difficulty* and between-subjects factor of *age group*. Moreover, three-way repeated-measures ANOVAs were used to analyze concomitant prefrontal activity (reflected through HbO2 concentrations), with within-subjects factor of task *difficulty*, between-subjects factor of *age group* and within-subjects factor of prefrontal *ROI*. Post-hoc contrasts were conducted using Fisher’s least significant difference (LSD) with Benjamini-Hochberg false discovery rate (FDR) multiple testing correction^82,83^. All results were considered significant at *p* < 0.05. One participant of the old group was excluded from all statistical analysis because its fNIRS data was not available due to a recording issue.

## Supplementary information

Supplementary information
