# Supplementary material for: Influences of age, mental workload, and flight experience on cognitive performance and prefrontal activity in private pilots: a fNIRS study
Source: Sci Rep. 2019 May 22;9:7688. doi: 10.1038/s41598-019-44082-w (PMC6531547; doi:10.1038/s41598-019-44082-w)
Supplement: Supplementary file 1 — Supplementary information [file 41598_2019_44082_MOESM1_ESM.doc]

**Influences of age, mental workload, and flight experience on cognitive performance and prefrontal activity in private pilots: a fNIRS study**

Mickaël Causse^1^, Zarrin K. Chua^2^ and Florence Rémy^2,3^

^1^ISAE-SUPAERO, Université de Toulouse, France

^2^Université de Toulouse, UPS, Centre de Recherche Cerveau et Cognition, France

^3^CNRS, Cerco, Toulouse, France

Address correspondence to:

Mickaël Causse,

ISAE-SUPAERO (DCAS),

10 avenue Edouard Belin,

BP 54032,

31055 TOULOUSE Cedex 4,

France

Tel. +33-5-61-33-81-28

Email: mickael.causse@isae.fr

**Supplementary Figures**


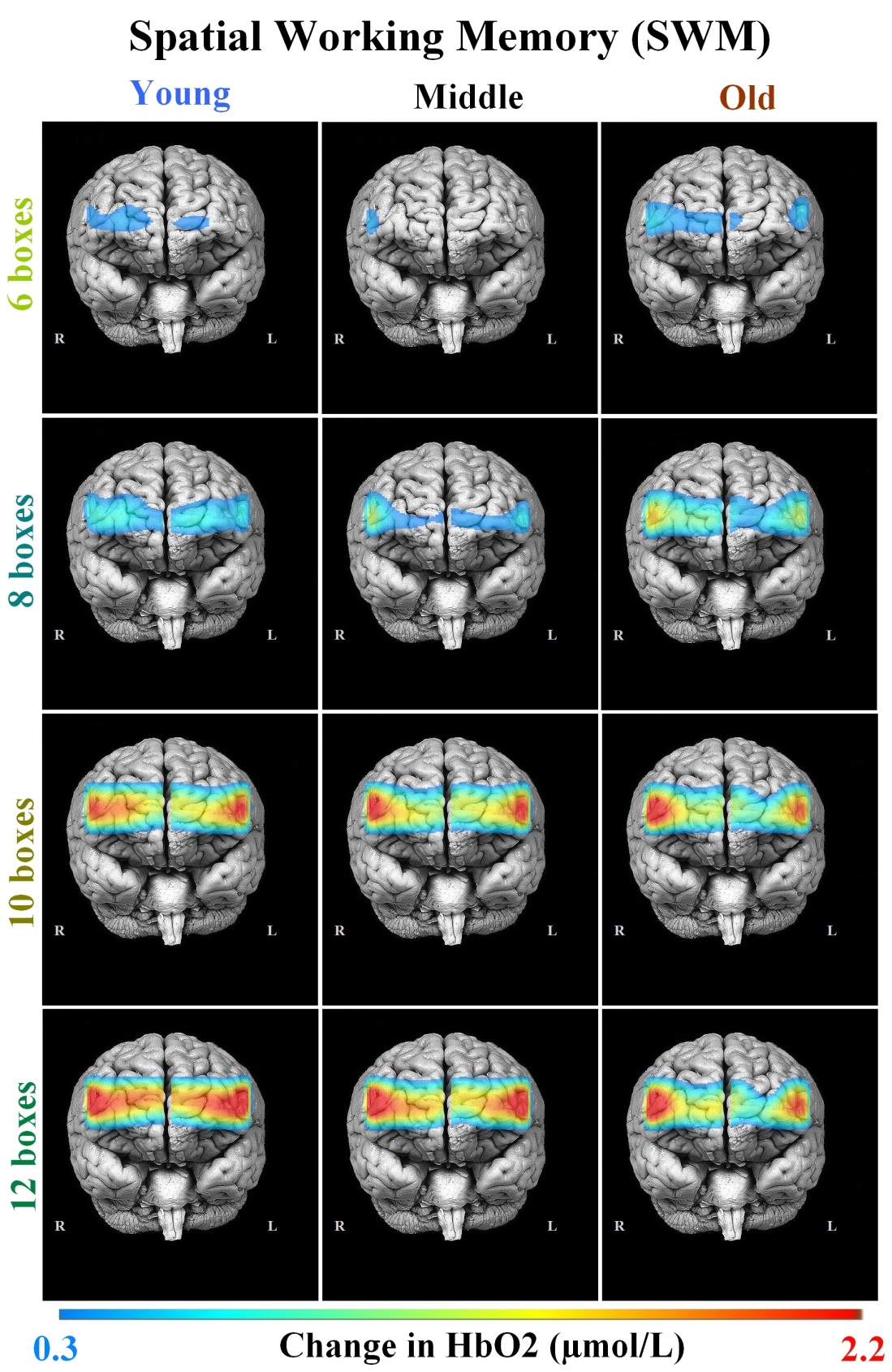


Supplementary Figure S1. Topographical map of the change in HbO2 concentration relative to rest (µmol/L) with respect to age groups across the four levels of difficulty of the spatial working memory task (SWM). Increased activation due to difficulty is observable across the four difficulty levels. Activation was more important in the lateral regions of the prefrontal cortex.


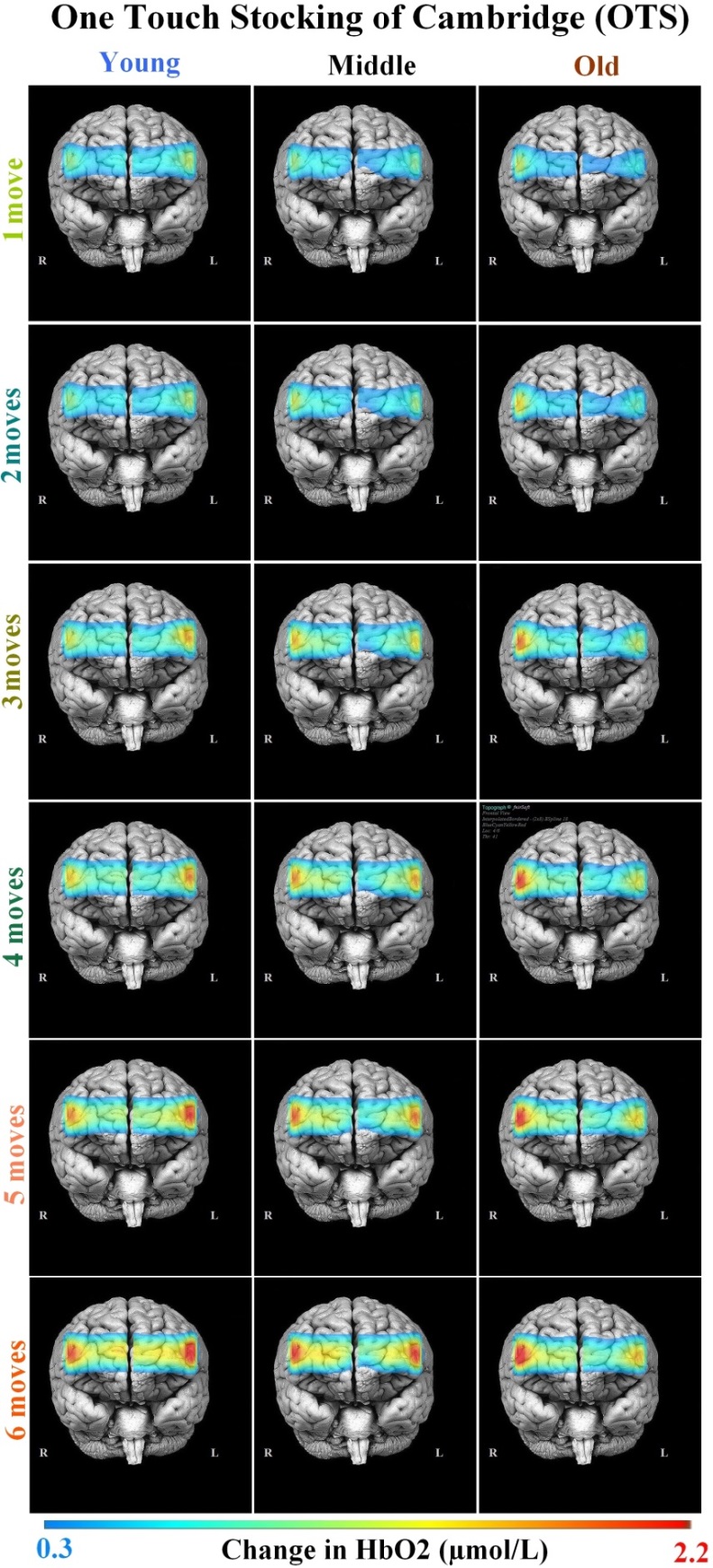


Supplementary Figure S2. Topographical map of the change in HbO2 concentration relative to rest (µmol/L) with respect to age groups across the six levels of difficulty of the One Touch Stocking of Cambridge task (OTS). Increased HbO2 is observable across the six levels of difficulty. Activation was more important in the lateral regions of the prefrontal cortex.

**Supplementary Tables**

Supplementary Table S1. Mean number of errors and standard error according to the level of difficulty with respect to age group during the Spatial Working Memory (SWM) task.

| **SWM** | 6 items | |  | 8 items | |  | 10 items | |  | 12 items | |
| --- | --- | --- | --- | --- | --- | --- | --- | --- | --- | --- | --- |
|  | *M* | *SE* |  | *M* | *SE* |  | *M* | *SE* |  | *M* | *SE* |
| Yng | 0.72 | 0.46 |  | 0.39 | 0.82 |  | 5.75 | 1.26 |  | 8.20 | 1.87 |
| Mid | 1.05 | 0.44 |  | 2.37 | 0.83 |  | 10.31 | 1.23 |  | 12.38 | 1.82 |
| Old | 2.78 | 0.40 |  | 4.26 | 0.75 |  | 16.59 | 1.012 |  | 20.02 | 1.66 |

Supplementary Table S2. Means and standard error for HbO2 and HHb concentration changes in the whole prefrontal cortex (16 voxels averaged) according to the level of difficulty with respect to age group during the Spatial Working Memory (SWM) task.

| **SWM** | 6 items | |  | 8 items | |  | 10 items | |  | 12 items | |
| --- | --- | --- | --- | --- | --- | --- | --- | --- | --- | --- | --- |
|  | *M* | *SE* |  | *M* | *SE* |  | *M* | *SE* |  | *M* | *SE* |
| Yng HbO2 | 0.26 | 0.10 |  | 0.51 | 0.16 |  | 1.41 | 0.20 |  | 1.71 | 0.19 |
| Yng HHb | -0.14 | 0.08 |  | -0.21 | 0.14 |  | -0.61 | 0.17 |  | -0.74 | 0.19 |
| Mid HbO2 | 0.11 | 0.10 |  | 0.39 | 0.16 |  | 1.32 | 0.19 |  | 1.59 | 0.18 |
| Mid HHb | -0.06 | 0.08 |  | -0.14 | 0.14 |  | -0.42 | 0.17 |  | -0.59 | 0.18 |
| Old HbO2 | 0.34 | 0.09 |  | 0.76 | 0.14 |  | 1.25 | 0.18 |  | 1.29 | 0.17 |
| Old HHb | -0.15 | 0.07 |  | -0.42 | 0.12 |  | -0.66 | 0.15 |  | -0.70 | 0.16 |

Supplementary Table S3. Mean number of errors (attempts) and standard error group according to the level of difficulty with respect to age during the One Touch Stockings (OTS) task.

| **OTS** | 1 move | |  | 2 moves | |  | 3 moves | |  | 4 moves | |  | 5 moves | |  | 6 moves | |
| --- | --- | --- | --- | --- | --- | --- | --- | --- | --- | --- | --- | --- | --- | --- | --- | --- | --- |
|  | *M* | *SE* |  | *M* | *SE* |  | *M* | *SE* |  | *M* | *SE* |  | *M* | *SE* |  | *M* | *SE* |
| Yng | 1.00 | 0.01 |  | 1.05 | 0.03 |  | 1.12 | 0.03 |  | 1.30 | 0.10 |  | 1.34 | 0.09 |  | 1.79 | 0.16 |
| Mid | 1.01 | 0.01 |  | 1.07 | 0.03 |  | 1.05 | 0.03 |  | 1.34 | 0.10 |  | 1.49 | 0.09 |  | 1.72 | 0.16 |
| Old | 1.04 | 0.02 |  | 1.10 | 0.04 |  | 1.14 | 0.03 |  | 1.548 | 0.08 |  | 1.54 | 0.08 |  | 2.11 | 0.14 |

Supplementary Table S4. Means and standard error for HbO2 and HHb concentration changes in the whole prefrontal cortex (16 voxels averaged) with respect to age group according to the level of difficulty with respect to age group during the One Touch Stockings (OTS) task.

| **OTS** | 1 move | |  | 2 moves | |  | 3 moves | |  | 4 moves | |  | 5 moves | |  | 6 moves | |
| --- | --- | --- | --- | --- | --- | --- | --- | --- | --- | --- | --- | --- | --- | --- | --- | --- | --- |
|  | *M* | *SE* |  | *M* | *SE* |  | *M* | *SE* |  | *M* | *SE* |  | *M* | *SE* |  | *M* | *SE* |
| Yng HbO2 | 0.63 | 0.15 |  | 0.64 | 0.16 |  | 0.85 | 0.17 |  | 0.96 | 0.18 |  | 1.14 | 0.19 |  | 1.30 | 0.19 |
| Yng HHb | -0.33 | 0.10 |  | -0.35 | 0.10 |  | -0.44 | 0.11 |  | -0.47 | 0.12 |  | -0.56 | 0.12 |  | -0.63 | 0.12 |
| Mid HbO2 | 0.60 | 0.15 |  | 0.65 | 0.15 |  | 0.85 | 0.17 |  | 0.98 | 0.17 |  | 1.06 | 0.18 |  | 1.21 | 0.19 |
| Mid HHb | -0.23 | 0.10 |  | -0.26 | 0.10 |  | -0.33 | 0.10 |  | -0.38 | 0.11 |  | -0.42 | 0.12 |  | -0.46 | 0.11 |
| Old HbO2 | 0.52 | 0.13 |  | 0.62 | 0.14 |  | 0.80 | 0.15 |  | 0.92 | 0.16 |  | 1.02 | 0.17 |  | 1.10 | 0.17 |
| Old HHb | -0.27 | 0.10 |  | -0.30 | 0.09 |  | -0.40 | 0.09 |  | -0.51 | 0.10 |  | -0.55 | 0.11 |  | -0.57 | 0.10 |
